# Efficacy of the iJobs Web-Based Psychoeducational Intervention to Improve Job Search Behavior and Promote Mental Health Among Unemployed People: Protocol for a Waitlist Randomized Controlled Trial

**DOI:** 10.2196/55374

**Published:** 2024-05-08

**Authors:** Alexandra Bodnaru, Andrei Rusu, Delia Vîrgă, Anja Van den Broeck, Roland W B Blonk, Loredana Marcela Trancă, Dragoș Iliescu

**Affiliations:** 1 Department of Psychology West University of Timișoara Timișoara Romania; 2 Department of Work and Organization Studies KU Leuven Brussels Belgium; 3 Optentia, North-West University Vanderbijlpark South Africa; 4 Department of Human Resource Studies Tilburg University Tilburg Netherlands; 5 Department of Social Work West University of Timișoara Timișoara Romania; 6 Department of Psychology and Cognitive Sciences University of Bucharest Bucharest Romania

**Keywords:** iJobs, JOBS II program, employability, internet intervention, randomized-controlled trial

## Abstract

**Background:**

Unemployment affects millions of people worldwide and, beyond its economic impact, has severe implications for people’s well-being and mental health. Different programs have been developed in response to this phenomenon, but to date, job-search interventions have proved to be most effective, especially the JOBS II program. The JOBS II program proved not only to be effective for re-employment but also has a positive impact on beneficiaries’ mental health (ie, reduces anxiety or depression). However, by now, this evidence-based program has been delivered only on site in the various countries where it was implemented. In the digital era, web-based alternatives to such programs are highly needed because they have the advantages of scalability and cost-effectiveness.

**Objective:**

In this context, we aim to investigate the efficacy of iJobs, the web-based adaptation of the JOBS II program, on job-search intensity and effort, the quality of job-search behaviors, and job-search self-efficacy. Further, 1 month after the intervention, we will also assess the employment status and the satisfaction with the job (if applicable). This study will also investigate the effect of iJobs on well-being and mental health (ie, anxiety and depression).

**Methods:**

This study is a 2-arm randomized controlled trial. The 2 independent groups (intervention vs waiting list control group) will be crossed with 3 measurement times (ie, baseline, the postintervention time point, and 1-month follow-up). The design will be a 2 (intervention vs control) × 3 (baseline, the postintervention time point, and 1-month follow-up) factorial design. iJobs is a 2-week intervention consisting of 6 modules: an introductive module and 5 modules adapted from the original JOBS II program to the web-based setting and Romanian population. The web-based intervention also has a human component, as beneficiaries receive personalized written feedback after each module on the platform from a team of psychologists involved in the project.

**Results:**

The enrollment of study participants started in June 2023 and is expected to end in May 2024. The data collection is expected to be completed by July 2024. The results are expected to be submitted for publication in the summer of 2024.

**Conclusions:**

This study is the first large-scale randomized controlled trial aiming to test the efficacy of a web-based adaptation of the JOBS II program. If our results support the efficacy of iJobs, they will offer the premise for it to become an evidence-based, accessible alternative for unemployed people in Romania and might be implemented in other countries.

**Trial Registration:**

ClinicalTrials.gov NCT05962554; https://clinicaltrials.gov/study/NCT05962554

**International Registered Report Identifier (IRRID):**

PRR1-10.2196/55374

## Introduction

### Background

Losing a job or not being able to find a job after finishing studies is a stressful life event—unemployed people struggle not only with financial strain but also with the stigma around it. Unemployment has become a vicious spiral that negatively affects individuals’ lives. As time passes, the motivation of unemployed people declines, which affects the intensity of the job-search process [1]. Hence, the chances of gaining re-employment decrease, and the well-being of unemployed people worsens. This can finally lead to mental health impairment (eg, depression and anxiety), and an essential moderator in this relationship is the duration of unemployment [2]. The alarming aspect of this phenomenon is not only how bad the impact of unemployment is at the individual level but also how many people are experiencing it worldwide.

In 2022, the unemployment rate was 6.1% in the European Union, but it is spread differently between countries, and more importantly, it is distributed asymmetrically within the same country regions. In Romania, the lowest percentage of unemployment is registered in the North-West and West regions (3.1% and 3.7%), whereas the highest rates range from 6.6% to 8.8% in the North-East and South regions [3]. From an economic perspective, the youth unemployment rate (people aged between 15 and 29 years) is more concerning. Romania’s Central and South regions register youth unemployment rates above 14.7%, and the European Union rate is 11.3% [3].

Various national programs and strategies are being developed to fight this phenomenon. However, the need for alternative solutions that promote employment (shortly after job loss) and well-being among unemployed people is evident. Efficient alternatives to classic governmental programs are job-search interventions. The JOBS II intervention [4] is one of them. JOBS is a job-search intervention intended to promote re-employment and mental health that differentiates itself from others by being studied in and adopted by many countries over the years, such as Finland [5], China [6], Ireland [7], Netherlands, Sweden, and South Korea [8], South Africa [9], or Germany [10].

Existing theories emphasize that unemployment is a cause of distress because unemployed people lose an essential social role (ie, status) and lack psychological basic needs fulfilled by one’s job (eg, time structure, social contact, activity, and financial security) [2]. Unsuccessful attempts to gain re-employment reduce the motivation of job seekers and affect their sense of competency and positive self-evaluations. In time, this can lead to mental health issues (ie, anxiety and depression). According to the results of a recent systematic review [11], JOBS improved participants’ self-esteem, self-efficacy (ie, job-search self-efficacy; JSSE), and ability to deal with setbacks (ie, inoculation against setbacks), which improved their well-being and mental health (on both the short and long term). Additionally, these coping mechanisms and job-search skills promoted and enhanced by JOBS are crucial for obtaining re-employment in a shorter period and obtaining a more qualitative job. Employment success and quality are predicted by job-search intensity and effort and job-search quality [12]. JOBS promotes job-search behaviors (JSBs; eg, beneficiaries make their job-search schedule) and the quality of those behaviors (eg, networking and practicing interviews).

The program’s effectiveness and popularity are based on its structure. It is a short-term intervention of 5 group sessions, encompassing almost all the components proven effective for job-search interventions [13]: *active learning, job-search skills, inoculation against setbacks, social support,* and *referent power.* During JOBS, participants learn the tasks they have to perform from the role of a job seeker and improve the necessary skills through group discussions, case studies, and role-playing exercises. They discover how to overcome potential setbacks in the re-employment process by brainstorming coping strategies, practicing their problem-solving skills, and receiving continuous social support from the trainers and beneficiaries.

However, JOBS is an on-site intervention that requires many resources to be delivered. This format restricts the number of beneficiaries and implies costs for both the organizers (eg, trainers and materials) and unemployed people to participate (eg, transportation fees). In the digitalization era and in the recent context of the COVID-19 pandemic that drastically changed the way we work and learn, it would be worthwhile to explore the efficacy of this intervention in a web-based format. An internet-delivered intervention can be an immediate solution at hand for those who just lost their jobs and could address more people at a time. Some possible advantages of such an alternative may also be the reduced costs and increased flexibility (ie, it is asynchronous).

iJobs is the first web-based adaptation of the JOBS II intervention, which was previously studied in a feasibility and acceptability trial [14]. The results were promising in terms of satisfaction with iJobs, so a further study testing its’ efficacy is worthwhile because it can provide information regarding the program’s impact on relevant outcomes.

### Specific Objectives

This study aims to investigate the efficacy of iJobs on job-search intensity and effort, the quality of JSBs, and JSSE. We will also assess the employment status and satisfaction with the job (for those who will be employed) 1 month after the intervention. This study will also investigate the effect of iJobs on well-being and mental health.

As a secondary objective, we will evaluate the moderating effects on re-employment of factors such as age, educational level, unemployment period, financial strain, and the mediating effects of job-search intensity, job-search quality, and self-efficacy on re-employment.

Since this is the first large-scale study on a web-based adaptation of the JOBS II program, as stated in the feasibility study [14], we will also evaluate the satisfaction with the intervention, the system usability, and the treatment adherence for the intervention groups.

Specifically, we will test the following hypotheses:

- H1: iJobs will increase the job-search intensity (H1a) and job-search effort (H1b) in the intervention group compared to the control group.
- H2: iJobs will increase the quality of job-search behaviors in the intervention group compared to the control group.
- H3: iJobs will increase job-search self-efficacy in the intervention group compared to the control group.
- H4: iJobs will increase self-esteem (H4a), inoculation against setbacks (H4b), and psychological capital (H4c) in the intervention group compared to the control group.
- H5: iJobs will decrease future career anxiety (FCA; H5a), depression (H5b), anxiety (H5c), and mental health complaints (H5d) in the intervention group compared to the control group.
- H6: The job-search intensity (H6a), job-search quality (H6b), and JSSE (H6c) will mediate the effect of iJobs on re-employment.
- H7: Age (H7a), educational level (H7b), unemployment period (H7c), and financial strain (H7d) will moderate the effect of iJobs on re-employment. iJobs will have stronger effects on re-employment for younger participants, participants with higher educational levels, participants with a shorter period of unemployment, and participants with lower levels of financial strain.

## Methods

### Ethical Considerations

Ethical approval regarding human subject research was obtained from the West University of Timișoara’s Scientific Council in May 2023 based on an application containing this study’s aim, procedure, measures, and materials (32005/17.05.2023). This study was registered as a clinical trial on ClinicalTrials.gov (NCT05962554). Participants will give their informed consent and, as per the European Union’s regulations, accept the General Data Protection Regulation statement when enrolling in the program: all the data from the questionnaires completed during and after the intervention is anonymous, their identity on the iJobs platform is protected by a random identification code that serves as the username, and they can withdraw from this study at any point. Participants who complete the follow-up measurements will be included in a raffle and can win either a backpack or an insulated bottle.

### Trial Design

This study is a 2-arm randomized controlled trial (RCT) that will test the efficacy of a web-based adaptation of iJobs. Participants will be randomly assigned (1:1 allocation) to either the intervention group, which benefits from the iJobs program, or a waiting list control group that will also receive the iJobs intervention 2 weeks after the intervention group finishes it. The 2 independent groups (intervention vs waiting list control group) will be crossed with 3 measurement times (ie, baseline, the postintervention time point, and 1-month follow-up). Hence, the design will be a 2 (intervention vs control) × 3 (baseline, the postintervention time point, and 1-month follow-up) factorial design.

### Participants and Recruitment

Eligible participants are unemployed Romanian adults. We will include in this study participants who (1) are unemployed and looking for a job, (2) work as volunteers and are looking for a paid job, (3) are aged between 18 and 60 years, and (4) have a PC or a laptop and basic digital skills. We will exclude the participants who do not have internet access or are lacking in availability during the 2-week program period.

Our recruitment strategy is to post an overview of iJobs on social media (ie, Facebook and LinkedIn), using paid advertising targeting job seekers, and on web-based newspapers. We will also promote iJobs on Employment Force Agencies and Recruitment Agencies across Romania.

### Intervention

**Figure 1:**
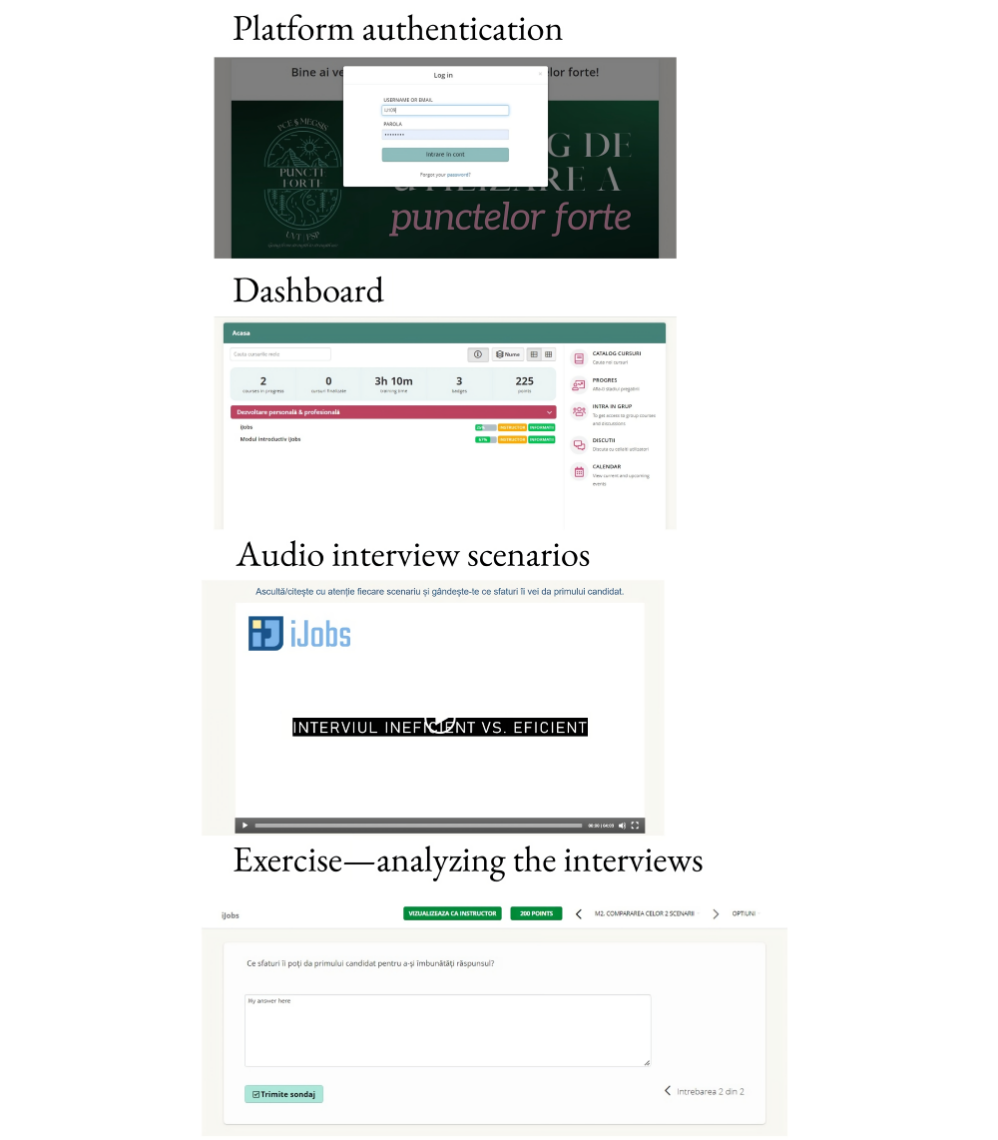
iJobs’ interface samples. From authentication to the main app dashboard, a preview of video material, and completing of an exercise on the platform.

iJobs is a web-based adaptation of the JOBS II program: *a manual for teaching people successful job-search strategies* [4]. iJobs is a 2-week intervention with 6 modules: an introductive module and 5 modules adapted from the original JOBS II program. iJobs will be delivered via TalentLMS (Epignosis), a web-based, cloud-based learning management system. iJobs’ visual interface is depicted in Figure 1. The intervention was previously tested in a feasibility and acceptability open-label trial using a different platform [14]. A brief description of iJobs modules can be found in Textbox 1. The program’s content is audio and text based, but the participants will answer in writing to all of the exercises.

iJobs is an asynchronous program, so the component of mutual social support between beneficiaries is absent. Compared to the JOBS II program, the participants interact via messages only with the counselors involved in the feedback process. The counselors were previously instructed about iJobs’ aim, content, and feedback process.

**Overview of iJobs modules. Brief description of each module’s content.**

**Introductive module**

- Participants will watch a short video explaining the platform’s main features (eg, how to complete exercises during the program and send messages to the counselors). At the end of the module, the participants must complete a questionnaire (eg, the pretest measures for the intervention group) to be enrolled in the intervention.

**Discovering your job skills**

- Participants will identify their strengths and learn how to demonstrate them during interviews from audio case studies that illustrate effective versus ineffective interviews.

**Dealing with obstacles to employment**

- Participants will identify possible obstacles and challenges in the employment process and explore how they can address them during interviews.

**Finding job openings**

- Participants will learn about networking and how their extended network can help identify job opportunities (eg, they complete an informational interview exercise).

**Resumes, contacts, and interviewing**

- The fourth module focuses on resumes (participants will analyze a resume and then create their curriculum vitae) and includes interview exercises from both the role of the candidate and the employer.

**Complete interview and planning for setbacks**

- Participants will integrate the information learned during the program into a complete interview. Ultimately, they will anticipate obstacles and prepare strategies for overcoming them in the re-employment process.

### Procedure

**Figure 2:**
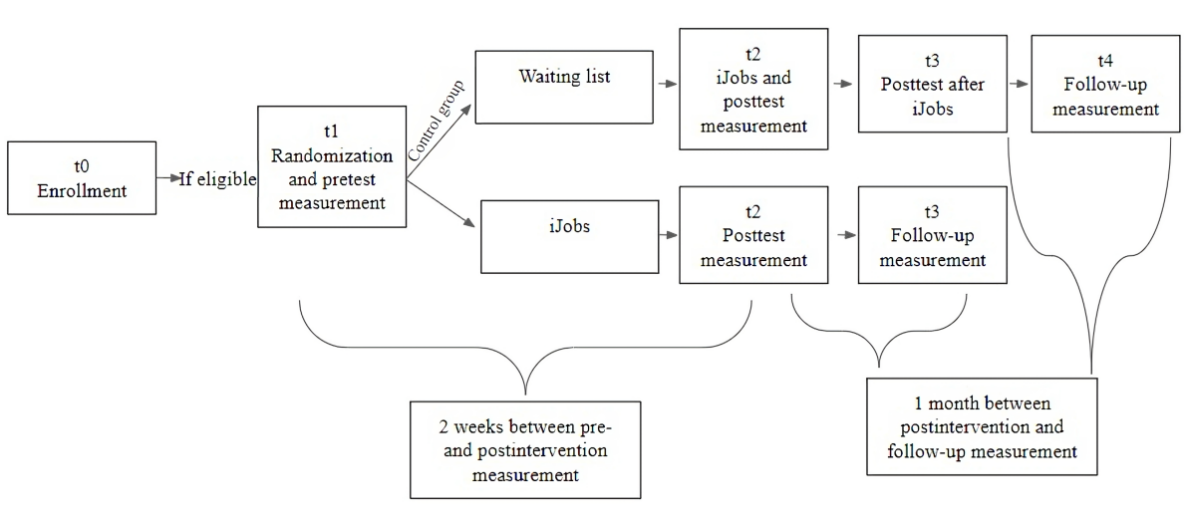
Study design and procedure overview. t0: 2-3 weeks before iJobs; t1: randomization within 1 week before iJobs and pretest assessments for both groups; t2: posttest assessments within 1 week after iJobs for both groups; the control group starts now iJobs; t3: follow-up screening 1 month after iJobs for the intervention group; posttest assessments for the control group at the end of iJobs; t4: follow-up screening 1 month after iJobs for the waiting list group.

The study procedure is presented in Figure 2. In the enrollment phase, an overview of the program and a link to open the iJobs presentation website [15] will be posted on social media (ie, paid advertising on Facebook and LinkedIn, web-based newspapers, and employment agency sites). Those who want to participate will have to complete a registration form, give their informed consent, and accept the General Data Protection Regulation statement. Eligible participants will be randomly allocated to either the intervention or the waiting list control group (1:1 allocation).

The participants from the intervention group will receive an email with the iJobs calendar and brief instructions. Afterward, they will receive an email with the platform login information. The username will be a random combination of digits (eg, IJ105) to ensure participants’ anonymity during the program. The account password is generated automatically by the platform. Once participants access their accounts, an introductive module containing the baseline measures questionnaire will be available on the platform. Meanwhile, the waiting list control group participants will receive an email with the iJobs period and a link to the baseline questionnaire.

On the iJobs’ platform, each module will be available for completion for 27 hours. After participants receive personalized written feedback on the platform from the team of counselors involved in the program, the following module will become automatically available for completion. After the last module, the posttest questionnaires will be available on the platform for 48 hours.

Further, 2 weeks after the intervention group finishes iJobs, the control group will start the intervention and go through the abovementioned steps. The questionnaire from the introductive module is the posttest measure for the control group.

Additionally, 1 month after the intervention, the participants who complete the posttest measures will be contacted to complete a brief questionnaire that evaluates their employment status and JSBs.

An overview of this study’s period is presented in Table 1.

**Table 1:** Study timeline. Time points and outcome assessments for the intervention group and waiting list group from enrollment to follow-up.

			Time point^a^								
			Enrollment (t0)		Randomization and pretest measurement (t1)		Posttest measurement (t2)		Follow-up intervention group (t3)		Follow-up control group (t4)
Enrollment			✓								
Eligibility screen			✓								
Informed consent			✓								
Randomization					✓						
Intervention group					✓		✓				
iJobs (intervention group)					✓						
Waiting list control group					✓		✓				
iJobs (control group)							✓				
**Assessments^b^**											
	Job-search intensity and effort			✓		✓		✓		✓	
	Job-search quality			✓		✓		✓		✓	
	Job-search self-efficacy			✓		✓		✓		✓	
	Self-esteem			✓		✓					
	Inoculation against setbacks			✓		✓					
	Psychological capital			✓		✓					
	Future career anxiety			✓		✓					
	Depression			✓		✓					
	Anxiety			✓		✓					
	Mental health complaints			✓		✓					
	Physical health	✓									
	Financial strain	✓									
	Sociodemographics	✓									
	Treatment adherence					✓		✓			
	Treatment satisfaction					✓		✓			
	System usability					✓		✓			
	Employment status	✓						✓		✓	
	Job quality (if applicable)							✓		✓	

^a^t0: 2-3 weeks before iJobs; t1: randomization within 1 week before iJobs and pretest assessments for both groups; t2: posttest assessments within 1 week after iJobs for both groups; the control group starts iJobs; t3: follow-up screening 1 month after iJobs for the intervention group; posttest assessments for the control group at the end of iJobs; t4: follow-up screening 1 month after iJobs for the waiting list group.

^b^For a detailed description, see the *Outcomes* section.

### Outcomes

#### Primary Outcomes

##### Job-Search Intensity and Effort

We will use a contemporary version of Blau’s JSB scale, adapted to the Romanian context [16], to assess the frequency and intensity of participants’ job-search activities during the past 2 weeks. The scale consists of 16 items on a 5-point scale (1=never; 5=very frequent). Thus, 13 items measure job-search intensity (eg, “How often did you send your CV to potential employers?”), with a total score between 13 and 65, and 3 items measure job-search effort (eg, “You dedicated much of your time to job search.”), with a total score between 3 and 15. A higher score means a better outcome. JSBs will be measured at baseline, the postintervention time point, and follow-up.

##### Job-Search Quality

We will use the Job Search Quality scale [17] to assess the quality of the job-search process, with precisely 4 components: goal establishment and planning, preparation and alignment, emotion regulation and persistence, and learning and improvement. The scale has 20 items on a 5-point scale (1=not at all applicable to me; 5=fully applicable to me). Goal establishment (ie, job-search goals and planning are clear, specific, and systematic) is measured with 7 items (eg, “I was determined to find a job.”); the total score ranges between 7 and 35. Preparation and alignment (ie, preparation of job-search activities in alignment with what the organizations are looking for in their applicants) is measured with 5 items (eg, “I carefully studied the website of organizations where I was going to apply.”); the total score ranges between 5 and 25. Emotion regulation (ie, self-control facilitating persistence in the job-seeking process; eg, “I persisted in my job search, even though it was unpleasant at times.”) and learning (ie, reflection on the job search and ways to improve it; eg, “I thought about other ways to find a job beyond those I had already tried.”) are measured with 4 items; the total score ranges between 4 and 20. A higher score means a better outcome. Job-search quality will be calculated at baseline, the postintervention time point, and follow-up.

##### About JSSE

The JSSE [18] will be used to assess participants’ perception of their ability to gain employment. The scale has 20 items (eg, “I believe my job search will be successful”) on a 5-point scale (1=a little; 5=a great deal), with a total score between 20 and 100. A higher score means a better outcome. JSSE will be measured at baseline, the postintervention time point, and follow-up.

#### Secondary Outcomes Measures

##### Inoculation Against Setbacks

Inoculation against setbacks will be assessed using 2 items (eg, “Do you have an action plan for potential setbacks in the re-employment process?”), on a 5-point Likert scale (1=completely disagree; 5=completely agree), retrieved from Vuori and Vinokur [5], aiming to measure the participants’ ability to deal with setbacks in the job-seeking process. The minimum score is 5, and the maximum is 10. A higher score means a better outcome. Inoculation against setbacks will be measured at baseline and the postintervention time point.

##### Self-Esteem

Rosenberg’s [19] Self-Esteem Scale will be used to assess global self-worth by measuring both positive and negative feelings about the self. The scale has 10 items (eg, “I believe I have many qualities.”) on a 4-point scale (1=completely disagree; 4=completely agree). The minimum score is 10, and the maximum is 40. A higher score means a better outcome. Self-esteem will be measured at baseline and the postintervention time point.

##### About FCA

The FCA scale [20] will be used to assess participants’ anxiety regarding their future jobs. The scale has 5 items (eg, “I worry about future employment because of fierce competition in the job market.”) on a 5-point scale (1=completely disagree; 5=completely agree), with scores between 5 and 25. A higher score means a worse outcome. FCA will be measured at baseline and the postintervention time point.

##### Anxiety

The Generalized Anxiety Disorder–7 assessment [21] will be used to assess the severity of anxiety symptoms in the past 2 weeks, according to the *Diagnostic and Statistical Manual of Mental Disorders, Fifth Edition* criteria. The scale has 7 items (eg, “Over the past two weeks, how often you had trouble relaxing?”) on a 4-point scale (0=not at all; 3=nearly daily), with scores between 0 and 21. A higher score means a worse outcome. Anxiety will be measured at baseline and the postintervention time point.

##### Depression

Patient Health Questionnaire–9 [22] will be used to measure participants’ severity of depression symptoms in the past 2 weeks, according to *the Diagnostic and Statistical Manual of Mental Disorders, Fourth Edition* criteria. The scale has 9 items (eg, “Over the last two weeks, how often you had little interest or pleasure in doing things?”) on a 4-point scale (0=not at all; 3=nearly daily). The minimum score is 0, and the highest one is 27. A higher score means a worse outcome. Depression will be measured at baseline and the postintervention time point.

##### Mental Health Complaints

We will use the Mental Health Complaints Scale [23], a 5-item instrument (eg, “In the past two weeks, how often were you happy?”) with a 6-point scale (1=not at all; 6=all the time). The minimum score is 6 and the maximum is 30. A higher score means a worse outcome. Mental health complaints will be measured at baseline and the postintervention time point.

##### Psychological Capital

The Compound Psychological Capital Scale [24] will be used to measure participants’ psychological capital. The scale has 12 items (eg, “Right now, I see myself as being pretty successful.”) on a 6-point Likert scale (1=completely disagree; 6=completely agree), with a minimum score of 12 and a maximum of 72. A higher score means a better outcome. Psychological capital will be measured at baseline and the postintervention time point.

#### Other Measures

##### Physical Health

A short version of Ware’s Physical Health Complaints Scale [25] will be used to assess participants’ physical health. The scale has 4 items (eg, “I get sick easier than other people.”) on a 5-point scale (1=completely disagree; 5=completely agree). The minimum score is 5 and the maximum is 25. A higher score means a worse outcome. Physical health will be measured only at baseline.

##### Sociodemographics

At baseline, participants will fill in their age, gender, residential area, educational level, average monthly income, unemployment period, work experience, and targeted professional field.

##### Treatment Adherence Measures

We will assess the dropout rate, the number of completed modules, and the quality of the completed assignments. Two independent experts will rate the degree of completeness and depth of the answer for each assignment based on an a priori grid (eg, “The participant understood the assignments.”). Treatment adherence will be measured at the postintervention time point.

##### Satisfaction With the Intervention

Satisfaction with the intervention will be measured using 21 items. The scale consists of 5 items (eg, “My trainer is competent.”) retrieved from a questionnaire used for measuring the alliance between trainers and trainees in a face-to-face JOBS intervention [26], and 9 items (eg, “Overall, how satisfied are you with the program?”) and 7 open-ended questions retrieved from Richards et al [27] and previously used in questionnaires evaluating the satisfaction with interventions delivered on e-cbt [28], the platform we previously used to provide the program. Satisfaction with the intervention will be measured at postintervention time point.

##### Usability

The System Usability Scale [29] will be used to measure participants’ satisfaction with TalentLMS, the web-based platform we will use for program delivery. The scale has 10 items on a 5-point scale (eg, “I think I would like to use this platform frequently”; 1=completely disagree and 5=completely agree); the calculation formula suggested by Brooke [30] generates a score between 0 and 100. A higher score means a better outcome. Usability will be measured at the postintervention time point.

##### Employment Status

We will assess the self-reported employment status with a dichotomous question at follow-up (ie, “Do you currently have a paid job?”).

##### Job Quality

The job quality will be measured at follow-up only for the participants who found employment, with 4 questions (a dichotomous question and 3 questions on a 10-point Likert scale; eg, “How much do you like your current job?”; 1=not at all and 10=very much). Specifically, we will assess the satisfaction with the professional domain, overall tasks, and salary.

### Statistical Analysis

#### Power

**Figure 3:**
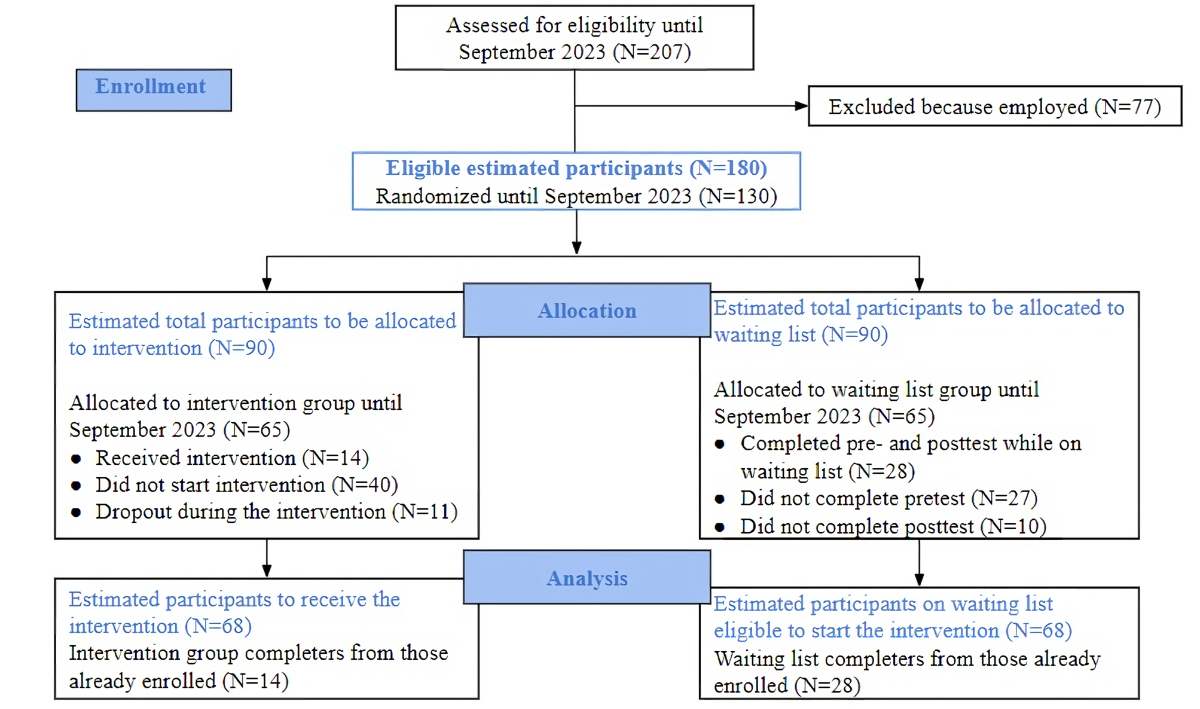
CONSORT flow diagram with the estimated number of participants aimed by the end of data collection and the current status of enrolled participants. CONSORT: Consolidated Standards of Reporting Trials.

The sample size estimation in GPower (Heinrich-Heine-Universität Düsseldorf) for a mixed factorial design aiming for a .80 statistical power is 132 participants. Based on our previous results from a feasibility and acceptability trial on iJobs, we can anticipate a possible dropout rate of about 36% (n=48). Thus, the final sample aimed for enrollment will be 180 eligible participants (see Figure 3 for the flowchart).

#### Data Analysis

We will use data from all the participants eligible for this study who completed the pretest measures. The data will be analyzed using the intent-to-treat framework with linear mixed model procedures. Intention-to-treat is based on the principle “once randomized, always analyzed.” It is a pragmatic approach to avoid bias in estimating the effect of treatment assignment in RCTs. We will use the linear mixed model approach without ad hoc imputations because it is more powerful than other options for studies with a high percentage of missing data [31].

For all the outcomes, time (baseline vs the postintervention time point) will be set as the within-group factor, and trial condition (intervention vs waiting list control group) will be used as the between-groups factor. We will analyze these 2 variables together with the entire set of outcomes (baseline vs postintervention comparison for the primary and secondary outcomes) to estimate the intervention’s effect size. Baseline to 1-month follow-up comparisons will be made to test how much the primary outcomes are preserved in time. We will also conduct separate analyses for each outcome with group, time, and group-by-time interaction as fixed effects and a random intercept for subjects with an identity covariance structure. The group-by-time interactions express the mean outcome change between the 2 trial groups from baseline to posttreatment.

## Results

The enrollment of study participants started in June 2023 and is expected to end in May 2024. Until September 2023, we organized 2 iJobs sessions. Out of the 130 randomized participants, we collected complete data (ie, pre- and postintervention) from 42 participants (*n*_intervention group_=14, *n*_control group_=28). The dropout rate from pretest to posttest in the intervention group was 44% (11/25) and 26% (10/38) in the waiting list control group (see Figure 3 for a flowchart). The data collection is expected to be completed by July 2024. The results are expected to be submitted for publication in the summer of 2024.

## Discussion

### Summary

This study aims to test the efficacy of iJobs, a web-based alternative to the JOBS II program, in the Romanian context and population. To our knowledge, this is the first large-scale RCT testing of the program’s efficacy as a web-based intervention. iJobs intends to increase re-employment and prevent mental health issues among its beneficiaries. Such an alternative for people experiencing unemployment is highly needed, mainly because, at the moment, there seem to be no available effective digital mental health interventions tailored to them [32]. Moreover, a web-based intervention should be easily accessible and cost-reduced, so that many unemployed people could benefit from it shortly after losing their jobs. This aspect is essential since the longer the unemployment period is, the worse the impact on an individual’s well-being [2].

### Limitations

Even if the web-based format of iJobs has some advantages, it also has some limitations. Social support is one of the most critical components of the JOBS II program. In the web-based format, mutual social support between beneficiaries is absent, while the support from the trainers comes only through written feedback messages. Immediate feedback for some types of exercises (eg, practice interviews) might be more valuable for participants. In addition, the solution for substituting social support constrains the participants to stick to specific timeframes while completing iJobs.

Another significant limitation is this study’s design. The waiting list control group also completes iJobs, so we cannot compare the groups regarding employment at the follow-up. The relatively high dropout rate is also a concerning factor. The recruitment strategy should be reconsidered to reduce the number of iJobs sessions taken and simultaneously increase the number of participants who benefit from them. By now, the program has been promoted on social media (ie, Facebook) through paid advertising. For further iJobs sessions, we are considering promoting the program also via web-based newspapers, recruiting agencies, and labor force agencies.

### Conclusions

If our results confirm iJobs’ efficacy, the intervention will have the perspective of becoming an accessible, evidence-based web-based solution for unemployed people in Romania. Moreover, it might be of interest to implement iJobs in other countries.

## Abbreviations

CV: curriculum vitae
FCA: future career anxiety
JSB: job-search behavior
JSSE: job-search self-efficacy
RCT: randomized controlled trial

## Data Availability

The data sets generated or analyzed during this study are not publicly available yet because the data collection is still ongoing, but preliminary data sets are available from the corresponding author upon reasonable request.
